# Biomechanics of transverse axis of medial longitudinal arch of children's foot based on 3D scanning

**DOI:** 10.3389/fped.2023.1197439

**Published:** 2023-07-10

**Authors:** Qinglin Liu, Chen Zhao, Xiaoxuan Yang, Jing Tang, Jing Chen, Li Tang, Jun Wu

**Affiliations:** ^1^ The First College of Clinical Medicine, Chongqing Medical University, Chongqing, China; ^2^ Department of Orthopaedics, The First Affiliated Hospital of Chongqing Medical University, Chongqing, China; ^3^ Shanqi (Chongqing) Smart Medical Technology Co. Ltd., Chongqing, China; ^4^ Department of Orthopaedics, Children's Hospital of Chongqing Medical University, National Clinical Research Center for Child Health and Disorders, Ministry of Education Key Laboratory of Child Development and Disorders, Chongqing Key Laboratory of Pediatrics, Chongqing, China

**Keywords:** paediatrics, biomechanics, foot, 3D scanning, transverse axis of arch

## Abstract

**Objective:**

To explore the application value of 3D scanning to obtain the parameters of transverse axis of medial longitudinal arch of foot in the biomechanical evaluation of transverse axis of medial longitudinal arch of foot in children.

**Method:**

The feet of children with flat foot, normal foot and high arched foot were scanned with the Foot Secret 3D scanner in the sitting and standing positions. The scanning data were imported into CATIA v5 software for measurement, to obtain four parameters of transverse axis of medial longitudinal arch from transverse arch angle, external transverse arch angle, curvature and transverse arch cross-sectional area.

**Result:**

There were statistically significant difference in transverse arch angle, external transverse arch angle and cross-sectional area between sitting and standing positions (*p* < 0.05). There were statistically significant differences in transverse arch angle, external transverse arch angle, curvature and transverse arch cross-sectional area among children with flat foot, normal foot and high arch foot (*p* < 0.05).

**Conclusion:**

The four parameters of transverse arch angle, external transverse arch angle, maximum curvature and cross-sectional area obtained by three-dimensional scanning can detect the changes of transverse axis of children's foot arch in different body positions with different foot types, which can be effectively used for the biomechanical evaluation of transverse axis of children's foot arch.

## 1. Introduction

The foot arch has a three-dimensional anatomical structure with complex and delicate motor function (1). The weight bearing and pressure buffering of the human body depend on the normal biomechanical characteristics of the foot arch. The abnormal changes of the foot arch structure inevitably lead to the biomechanical changes of the lower limbs, causing musculoskeletal injuries and diseases of the lower limbs, and affecting the motor function and quality of life to varying degrees (2). Research shows that the abnormal development of children's foot arch may affect gait and motor function, lead to abnormal force line of lower limbs, and cause bone and joint disease of lower limbs (3, 4). The detection and evaluation of the development of children's foot arch is helpful to clarify the pathological mechanism of the injury and disease related to the abnormal development of foot arch, and has great significance for clinical decision-making.

At present, the evaluation of children's foot arch development mainly depends on physical examination (5), imaging examination (6) and foot print examination (7). The clinical physical examination is heavily dependent on the clinical experience of the examiner. Imaging examination has the problems of radiation, etc (8). Footprint examination only reflect the information of axial plane, and cannot accurately predict the height of sagittal plane, or truly reflect the shape of foot arch, which brings low diagnostic sensitivity (9).

Biomechanical research of foot arch can provide a lot of useful information for studying the structure, function and posture control of foot arch (10). Generally, the research of foot biomechanics mostly adopts finite element model simulation research, but the finite element simulation analysis method can only be a useful supplement and verification of experimental biomechanical research, and the authenticity of its conclusions ultimately needs to be tested by experimental research (11, 12). Based on the force-measuring sensor technology, researchers have developed flat-plate, insole-type and sensor-type foot dynamics testing systems, which are characterized by high efficiency, flexibility, mobility and low cost, paving the way for the development of foot biomechanical testing systems (13, 14). By measuring the pressure between the sole and the contact surface, we can provide objective and dynamic reference for understanding the structure and function of the foot.

The arch of the foot includes the internal and external longitudinal arch and the transverse arch of the middle and anterior foot. The structure, pathology and clinical significance of the internal and external longitudinal arch have been deeply studied, while the transverse axis of arch (TAA) has been relatively less studied (15). The foot TAA forms an arched elastic structural system with very reasonable mechanical properties in the foot, protects the nerves and blood vessels of the foot from compression, and plays an important role in absorbing shock and buffering the stress of the foot (16). Studies have shown that the collapse of foot TAA is prone to irregular pain and painful callose of the foot. The deformation of foot TAA is closely related to many foot diseases in children (17, 18). Puszczalowska-Lizis, Ewa et al. reported that reduction of the transverse arch of the foot has an effect on the severity of fifth toe flexion, leading to disorders of the middle phalangeal joint (17). Therefore, biomechanical evaluation and detection of the development of children's foot TAA has important clinical significance.

In this study, the feet of children with flat foot, normal foot and high arched foot were scanned by 3D to obtain four arch parameters: transverse arch angle, external transverse arch angle, curvature and cross-sectional area, and to explore the application value of these four parameters in the biomechanical evaluation of children's TAA.

## 2. Method

This study was approved and supervised by the Ethics Committee of Children's Hospital Affiliated to Chongqing Medical University (batch number: 2019CR32). Children aged between 7 and 9 years having capability of cooperating with the test independently were included in the study. The exclusion criteria were: history of lower extremity trauma, fracture, surgery, neuromuscular dysfunction of lower extremity, limited joint movement of the foot, and rigid foot type. Based on the anteroposterior and lateral radiographs of both feet, which were diagnosed by pediatric orthopedic doctors with more than 10 years of professional work experience, participants were divided into flat foot group, normal foot group and high arch foot group, each group including 15 children. All participants and their guardians volunteered to participate in the study and signed informed consent before study.

**Figure 1:**
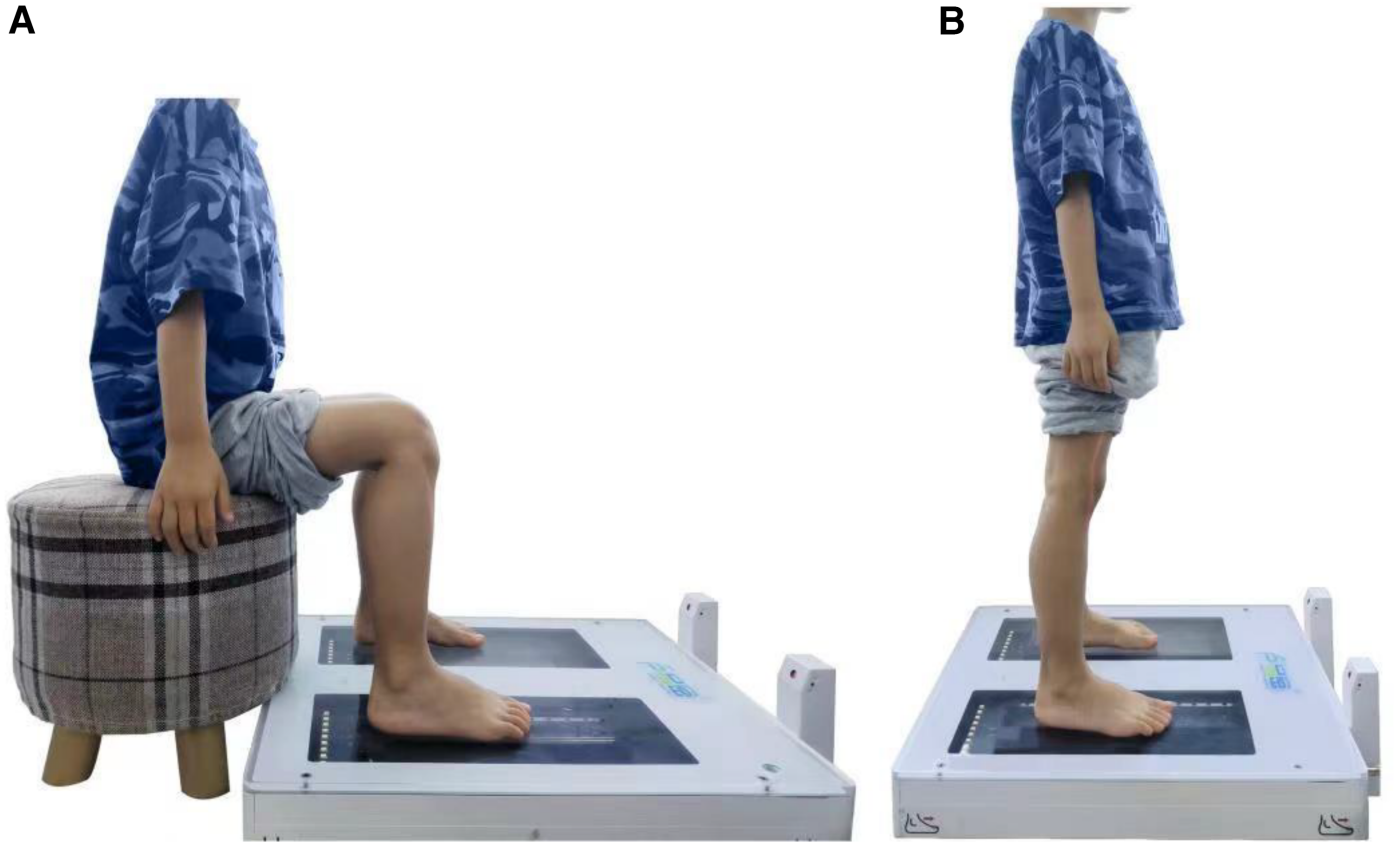
Foot scanning with the “Foot Secret 3D Scanner” in children. (**A**) Sitting positions, (**B**) standing positions.

The foot is scanned with the “Foot Secret 3D Scanner” produced by Shanqi (Chongqing) Intelligent Medical Technology Co., Ltd. The scanning method is as described in our previous article report (19). In short, all participants were measured in sitting and standing positions (Figure 1). During the sitting position measurement, the participants' hip joints and knee joints kept 90 ° flexion, the ankle joints were in a neutral position, and the second toe of the foot was aligned with the laser axis of the instrument (Figure 1A). During the standing position measurement, the participants stood naturally, with their feet the same width as their shoulders, and kept the acromial process, the center of the hip joint, the center of the knee joint and the lateral ankle aligned, and the second toe of the foot aligned with the laser axis of the instrument (Figure 1B). Scan each position three times.

**Figure 2:**
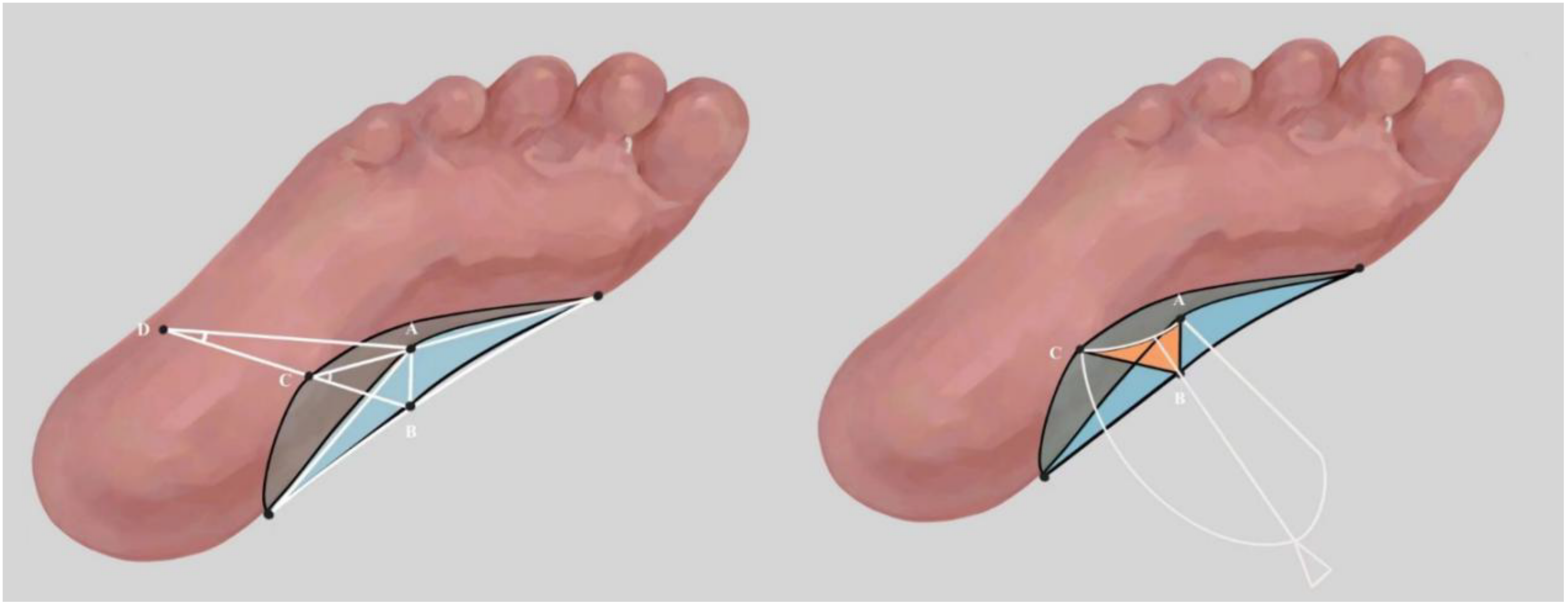
Schematic diagram of transverse axis of the arch measurement. (A) The highest point of the inner side of the arch volume, (B) the highest point of the inner side of the arch volume which is vertically projected on the transverse plane, (C) the outermost point of the arch volume, (D) the outermost point of the foot, ∠ ACB is the transverse arch angle, ∠ ADB is the external transverse arch angle, *κ* AC is the curvature of the transverse axis of the foot arch, and the area of the irregular figure ABC (orange part) is the cross-sectional area of the transverse arch of medial longitudinal.

In order to obtain the most accurate TAA parameters, the scanning data is imported into CATIA v5 software for measurement. In order to reduce the errors within and between observers, two experienced data analysts conducted two independent measurements of each parameter. Because of the three-dimensional characteristics of arch, the measurement of TAA parameters is analyzed on the basis of arch volume. The measured parameters include transverse arch angle, external transverse arch angle, transverse arch curvature and transverse arch cross-sectional area. Figure 2 is a schematic diagram of measurement. A is the highest point of the inner side of the arch volume, B is the highest point of the inner side of the arch volume that is vertically projected on the transverse plane, C is the outermost point of the arch volume, and D is the outermost point of the foot. The transverse arch angle is the included angle between AC line and BC line. The external transverse arch angle is the included angle between AD line and BD line. The curvature is the maximum curvature of the irregular line segment from point A to point C. The cross-sectional area of the transverse arch is the irregular area formed by points A, C and B.

Use R Studio (Version 1.2.5033 © 2009–2019 R Studio, Inc.) for statistical analysis. We use descriptive statistics, mean and standard deviation to analyze the data. Kolmogorov-Smirnov test was used to test the normality before data analysis. Univariate analysis of variance and Kruskal-Wallis test were used to compare the differences among flat foot group, normal foot group and high arch foot group. Student-Newman-Keuls Test is used for comparison between the three groups. The paired *t* test and Wilcoxon Signed Ranks Test were used to analyze the significant differences between sitting and standing positions. The significance level with *p* value less than 0.05 was used for all analyses.

## 3. Result

In this study, 15 children were included respectively in the flatfoot group, the normal foot group and the high arch foot group. The demographic characteristics of each group are shown in Table 1. There was no significant difference in age, height, weight and BMI among the groups (*p* > 0.05).

**Table 1:** Demographic characteristics.

		Flat foot	Normal foot	High arch foot	Total	*p*
Gender	Male	7	7	8	22	>0.05
	Female	8	8	7	23	
Age (years)		8.00 ± 0.79	8.23 ± 0.65	8.31 ± 0.74	8.18 ± 0.72	0.476
Height (cm)		127.93 ± 7.29	133.53 ± 7.99	134.03 ± 7.95	131.83 ± 8.08	0.069
Weight (kg)		28.03 ± 6.01	29.53 ± 5.51	28.95 ± 7.47	28.84 ± 6.27	0.812
BMI (kg/m^2^)		17.01 ± 2.83	16.42 ± 1.82	15.87 ± 2.15	16.44 ± 2.30	0.407

The measurement results of the TAA parameters of the left and right feet in the flatfoot group, the normal foot group and the high arch foot group are shown in Table 2. Since the difference of the TAA parameter of the left and right feet in each group is not statistically significant, the subsequent data analysis will not distinguish the left and right feet.

**Table 2:** Analysis of the transverse axis of arch parameters of left and right feet.

	Flat foot				Normal foot				High arch foot			
	左	右	t	p	左	右	t	*p*	左	右	t	*p*
Transversal arch angle (°)	17.95 ± 4.69	17.54 ± 5.69	0.22	0.829	19.24 ± 4.99	16.86 ± 6.48	1.13	0.270	14.89 ± 3.50	16.23 ± 5.56	−0.79	0.438
External transverse arch angle (°)	10.46 ± 2.04	9.65 ± 2.55	0.96	0.347	14.49 ± 2.06	13.40 ± 2.99	1.15	0.261	14.71 ± 2.85	14.90 ± 3.88	−0.16	0.876
curvature	0.03 ± 0.01	0.04 ± 0.02	−1.34	0.192	0.04 ± 0.02	0.05 ± 0.02	−1.10	0.279	0.06 ± 0.02	0.06 ± 0.02	0.03	0.975
Cross sectional area	96.34 ± 33.66	89.01 ± 41.06	0.54	0.597	165.25 ± 41.10	154.61 ± 35.89	0.76	0.456	204.89 ± 51.95	200.66 ± 55.64	0.22	0.831

The TAA parameters of the feet of children with flat feet, normal feet and high arched feet in sitting and standing positions are obtained by 3D scanning, and the results are shown in Table 3.

**Table 3:** The results of transverse axis of arch parameters of children with flat foot, normal foot and high arch foot in sitting and standing positions.

		Flat foot	Normal foot	High arch foot
Transversal arch angle (°)	Sitting position	17.74 ± 5.12	18.05 ± 5.81	15.56 ± 4.61
	Standing position	14.24 ± 5.37	16.13 ± 4.75	10.80 ± 3.23
External transversal arch angle (°)	Sitting position	10.06 ± 2.30	13.94 ± 2.59	14.80 ± 3.34
	Standing position	5.29 ± 1.56	9.32 ± 2.59	8.47 ± 2.76
Curvature	Sitting position	0.04 ± 0.01	0.04 ± 0.02	0.06 ± 0.02
	Standing position	0.04 ± 0.01	0.04 ± 0.02	0.05 ± 0.01
Cross sectional area	Sitting position	92.68 ± 37.08	159.93 ± 38.30	202.77 ± 52.94
	Standing position	37.11 ± 15.93	98.44 ± 25.49	127.34 ± 39.87

**Figure 3:**
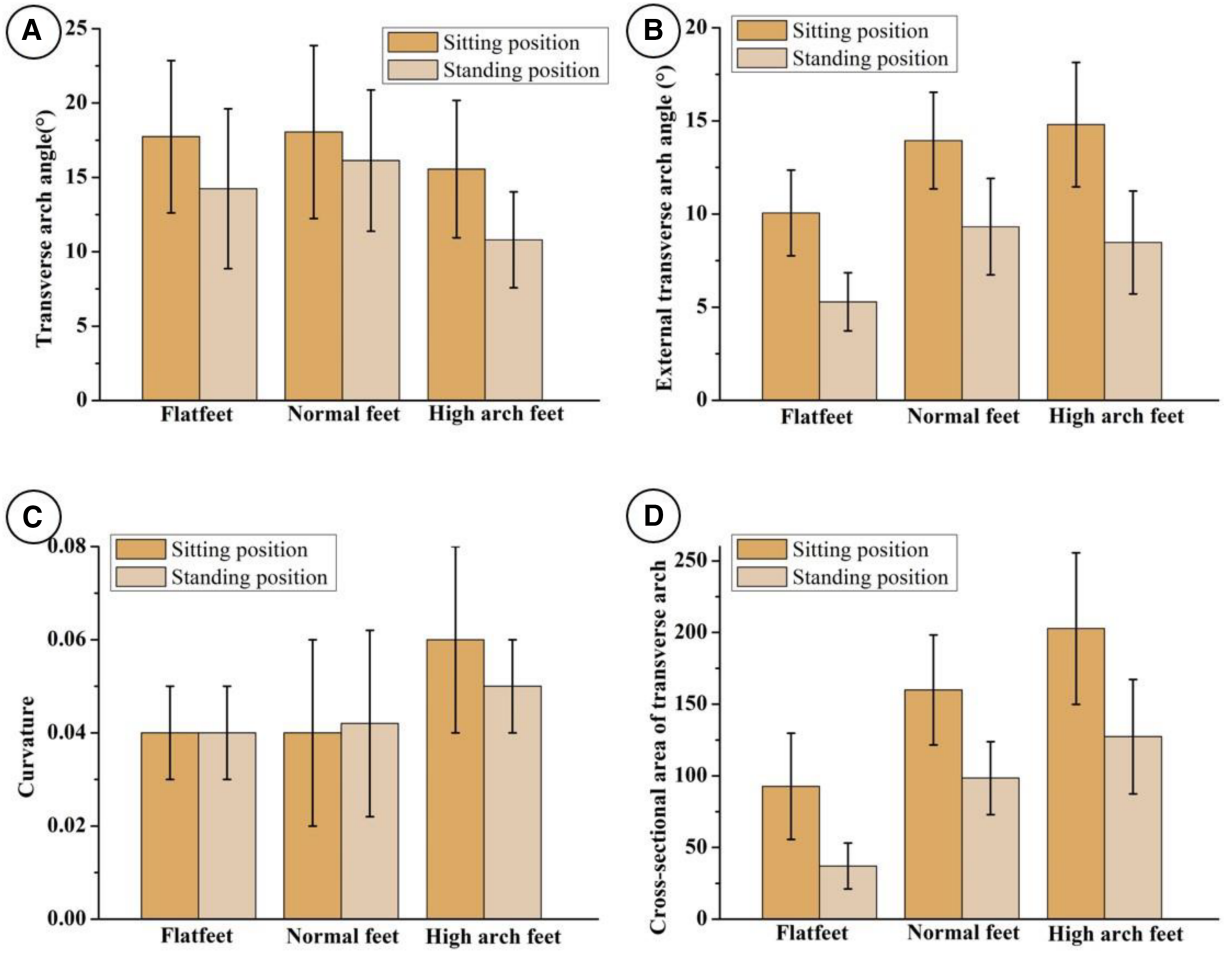
Measurement results of transverse axis of arch parameters of flat foot, normal foot and high arch foot in sitting and standing positions. (**A**) Transverse arch angle, (**B**) external transverse arch angle, (**C**) curvature, (**D**) cross-sectional area.

Compare the TAA parameters of the foot in the sitting and standing positions of different foot types, and the results are shown in Figure 3. The transverse arch angle of flat foot, normal foot and high arched foot were significantly different in sitting and standing positions (*p* < 0.05). Similarly, the external transverse arch angle of flat foot, the cross-sectional area of flat foot, the external transverse arch angle of normal foot, the cross-sectional area of normal foot, the external transverse arch angle of high arch foot and the cross-sectional area of high arch foot have statistically significant differences in sitting and standing positions (*p* < 0.05). The maximum curvature of the high arch foot has statistical significance in the sitting and standing positions (*p* < 0.05), but the curvature of the flat foot and the normal foot have no statistical significance in the sitting and standing positions (*p* > 0.05).

**Figure 4:**
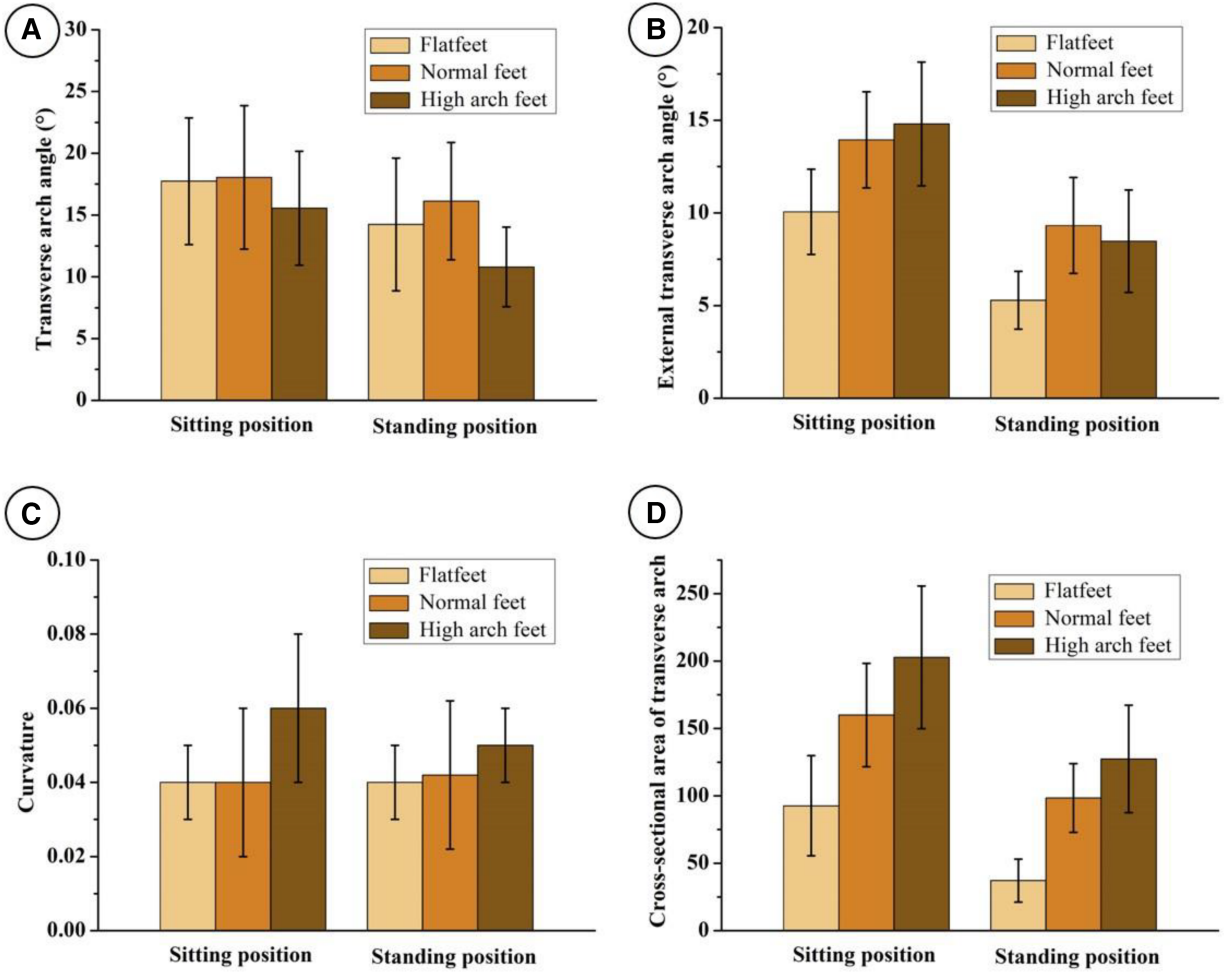
Measurement results of transverse axis of the arch parameters of flat foot, normal foot and high arch foot in sitting and standing positions. (**A**) Transverse arch angle, (**B**) external transverse arch angle, (**C**) curvature, (**D**) cross-sectional area.

Compare the TAA parameters of different foot types in sitting and standing positions, and the results are shown in Figure 4. In the sitting position, the difference of transverse arch angle between flat foot, normal foot and high arch foot was statistically significant (*p* < 0.05). Similarly, the difference of external transverse arch angle and transverse arch cross-sectional area in the three groups was statistically significant (*p* < 0.05). Compared with the curvature of flat foot and normal foot, the curvature of high arch foot has statistical significance (*p* < 0.05), but the difference between the transverse arch curvature of flat foot and that of normal foot has no statistical significance (*p* > 0.05). When standing, the difference of four transverse arch parameters of flat foot, normal foot and high arch foot was statistically significant (*p* < 0.05).

## 4. Discussion

With the popularization of 3D technology, 3D scanning, as a new way of foot biomechanical detection, can quickly obtain foot models and foot parameters, having the significant features of low cost, sensitivity and visual (20). As stated in our previous research, the volume of the foot arch obtained by the 3D scanner can effectively reflect the three-dimensional characteristics of the foot, including not only the vertical height of the medial longitudinal arch, but also the width and length of the medial longitudinal arch, which conforms to its three-dimensional characteristics. Moreover, by integrating the foot arch volume data under non-weight bearing position and weight bearing position, it can authentically reflect the dynamic shape changes of the medial longitudinal arch, with higher accuracy and sensitivity (19). Wang J et al. established the scale of foot arch volume development for children aged 3–12 years. The foot arch volume has become a new morphological measurement method of the medial longitudinal arch of the foot (21). On the basis of previous researches, this study further explores the application value of foot parameters obtained by 3D scanning in the detection and evaluation of children's transverse axis of arch (TAA) of foot.

In this study, the parameters of TAA of foot under different body positions were analyzed. The foot TAA is a relatively stable and elastic arch structure composed of cuboids, 3 cuneiform bones, 5 metatarsals and muscle ligaments. At present, most scholars believe that the three-dimensional structure of the TAA of foot changes with different body positions (22). The interaction of the first, fourth and fifth metatarsal heads forms the base of the TAA of the forefoot when not loaded, and the second and third metatarsal heads lift slightly upward to form an arch on the cross section. When loaded, the second, third and fourth metatarsal heads sink, the forefoot widens, and the TAA decreases (23). Kudo S, et al. evaluated the flexibility of the TAA of the forefoot by measuring the percentage of change in the length of the TAA of the forefoot at the standing position and the lower leg maximum anterior tilting position (24). Matsushita T et al. found that there was a significant difference in the height of the foot TAA with 90% of weight bearing and 10% of weight bearing (25). The transverse arch angle, external transverse arch angle and cross sectional area of the children tested in the standing position are reduced to different degrees compared with those in the sitting position, with statistical significance. The results are consistent with the literature reports. Therefore, the transverse arch angle, external transverse arch angle and cross-sectional area of the foot can be used to detect and evaluate the changes of children's transverse arch in different body positions.

The TAA of the foot and the internal and external longitudinal arch form an arched elastic structure system with very reasonable mechanical properties in the foot (26). The TAA allows the longitudinal arch to be flexible as a lever, and at the same time, the foot arch is rigid as a strong spring lever (1). The biomechanical properties of the internal and external longitudinal arch have been studied in depth, while the biomechanical properties of the TAA are relatively less (15). Therefore, this study uses 3D scanning to obtain the foot model and foot parameters, and then analyzes the TAA of the foot. In the sitting position, the differences of transverse arch angle, external transverse arch angle and transverse arch cross-sectional area of flat foot, normal foot and high arch foot were statistically significant (*p* < 0.05). In the standing position, the differences of transverse arch angle, external transverse arch angle, curvature and transverse arch cross-sectional area of flat foot, normal foot and high arch foot were statistically significant (*p* < 0.05). The results of this study show that four transverse arch parameters, including transverse arch angle, external transverse arch angle, curvature and transverse arch cross-sectional area, can be used to detect and evaluate the changes of TAA of children with different foot types.

At present, children's orthopedic surgeons' diagnosis and curative evaluation of foot deformities such as flatfoot and high-arched foot still mainly depend on the longitudinal arch of the foot, ignoring the TAA of the foot (17). Venkadsan M et al. found that the TAA of the foot can affect the flexibility of the longitudinal arch of the foot through the function of the soft tissue between the metatarsal bones. They believed that this discovery would help improve the clinical treatment of foot deformity and the research of foot motor function (1). Bardyugov PS et al. believed that in the process of treating foot deformities, reconstructing the structure of the TAA of the foot can achieve the effect of treatment and beauty, and improve the quality of patients' life (27). In this study, there are differences in the transverse arch parameters between flatfoot and high-arched foot and normal foot, indicating that there are not only changes in the longitudinal arch, but also changes in the transverse arch of flatfoot and high-arched foot. Therefore, we believe that the comprehensive analysis of the longitudinal arch parameters (foot arch volume) and the TAA parameters (transverse arch angle, external transverse arch angle, curvature and transverse arch cross-sectional area, etc.) obtained by three-dimensional scanning will help to accurately evaluate the development of children's feet and improve the clinical diagnosis and treatment efficacy evaluation of foot diseases such as flat feet and high arched feet. On the other hand, clarifying the measurement of the transverse arch and its contribution in exercise can provide clinical practitioners with complete information. And based on 3D-scanning technic, physicians and therapists can quickly and dynamically acquire MLA longitudinal and transverse axis parameters to provide a basis for guiding children's growth and development and clinical decision making. In the future, we will expand the sample size, establish a database of medial longitudinal arch longitudinal and transverse axis morphology, and establish a series of foot type diagnostic criteria based on 3D parameters.

In this study, four parameters, namely transverse arch angle, external transverse arch angle, transverse arch curvature and transverse arch cross-sectional area, were used to analyze the TAA of children's feet in different positions with different foot types. However, there are some shortcomings. First of all, due to the limited sample size, this study cannot accurately describe the changes of the TAA of children's flatfoot and high-arched foot. It is necessary to further expand the sample size to clarify the change pattern of the TAA of deformed foot. Second, only children aged 7–9 years old were included in this study. The age range needs to be further expanded to find out the development trend of children's TAA of foot.

## 5. Conclusion

In this study, four parameters of transverse arch angle, external transverse arch angle, curvature and cross-sectional area were obtained by 3D scanning, and the transverse axis of arch of children's feet under different body positions and different foot types were analyzed. The results showed that the transverse arch angle, external transverse arch angle and cross-sectional area can detect the changes of children's transverse axis of arch of foot in different body positions, and the transverse arch angle, external transverse arch angle, curvature and cross-sectional area can reflect the changes of children's transverse axis of arch of foot in different foot types. Therefore, the four parameters of transverse arch angle, external transverse arch angle, curvature and cross-sectional area obtained by three-dimensional scanning can be effectively used for the biomechanical evaluation of children's transverse axis of arch, which can provide an important reference for arch screening and treatment plan development.

## Data Availability

The original contributions presented in the study are included in the article, further inquiries can be directed to the corresponding authors.

## References

[1] Venkadesan M Yawar A Eng CM Dias MA Singh DK Tommasini SM Stiffness of the human foot and evolution of the transverse arch. Nature. (2020) 579(7797):97–100. 10.1038/s41586-020-2053-y 32103182

[2] Hatfield GL Cochrane CK Takacs J Krowchuk NM Chang R Hinman RS Knee and ankle biomechanics with lateral wedges with and without a custom arch support in those with medial knee osteoarthritis and flat feet. J Orthop Res. (2016) 34(9):1597–605. 10.1002/jor.23174 26800087

[3] Kothari A Dixon PC Stebbins J Zavatsky AB Theologis T. The relationship between quality of life and foot function in children with flexible flatfeet. Gait Posture. (2015) 41(3):786–90. 10.1016/j.gaitpost.2015.02.012 25771182

[4] Boryczka-Trefler A Kalinowska M Szczerbik E Stępowska J Łukaszewska A Syczewska M. Effect of Plano-Valgus foot on lower-extremity kinematics and spatiotemporal gait parameters in children of age 5-9. Diagnostics (Basel). (2021) 12(1):2. 10.3390/diagnostics12010002 35054169 PMC8774692

[5] Zuil-Escobar JC Martínez-Cepa CB Martín-Urrialde JA Gómez-Conesa A. Evaluating the medial longitudinal arch of the foot: correlations, reliability, and accuracy in people with a low arch. Phys Ther. (2019) 99(3):364–72. 10.1093/ptj/pzy149 30535273

[6] Vanderwilde R Staheli LT Chew DE Malagon V. Measurements on radiographs of the foot in normal infants and children. J Bone Joint Surg Am. (1988) 70(3):407–15. 10.2106/00004623-198870030-00013 3346265

[7] Kanatli U Yetkin H Cila E. Footprint and radiographic analysis of the feet. J Pediatr Orthop. (2001) 21(2):225–8. 10.1097/01241398-200103000-00018 11242255

[8] Buchberger B Scholl K Krabbe L Spiller L Lux B. Radiation exposure by medical x-ray applications. Ger Med Sci. (2022) 20:Doc06. 10.3205/000308 35465642 PMC9006309

[9] Ferri M Scharfenberger AV Goplen G Daniels TR Pearce D. Weightbearing CT scan of severe flexible pes planus deformities. Foot Ankle Int. (2008) 29(2):199–204. 10.3113/FAI.2008.0199 18315976

[10] Foulston J. Biomechanical analysis of foot structure and function. Baillieres Clin Rheumatol. (1987) 1(2):241–60. 10.1016/s0950-3579(87)80002-x 3331323

[11] Phan PK Vo ATN Bakhtiarydavijani A Burch R Smith B Ball JE In silico finite element analysis of the foot ankle complex biomechanics: a literature review. J Biomech Eng. (2021) 143(9):090802. 10.1115/1.4050667 33764401

[12] Lagravère M. Finite element analysis: is it justifiable? Am J Orthod Dentofacial Orthop. (2021) 159(3):255–6. 10.1016/j.ajodo.2020.10.017 33641812

[13] Hendriks MMS Vos-van der Hulst M Weijs RWJ van Lotringen JH Geurts ACH Keijsers NLW. Using sensor technology to measure gait capacity and gait performance in rehabilitation inpatients with neurological disorders. Sensors (Basel). (2022) 22(21):8387. 10.3390/s22218387 36366088 PMC9655369

[14] Homes R Clark D Moridzadeh S Tosovic D Van den Hoorn W Tucker K Comparison of a wearable accelerometer/gyroscopic, portable gait analysis system (LEGSYS+TM) to the laboratory standard of static motion capture camera analysis. Sensors (Basel). (2023) 23(1):537. 10.3390/s23010537 36617135 PMC9824443

[15] Babu D Bordoni B. Anatomy, bony pelvis and lower limb, medial longitudinal arch of the foot. In: Statpearls. Treasure Island (FL): StatPearls Publishing. (2022). 32965960

[16] Asghar A Naaz S. The transverse arch in the human feet: a narrative review of its evolution, anatomy, biomechanics and clinical implications. Morphologie. (2022) 106(355):225–34. 10.1016/j.morpho.2021.07.005 34419345

[17] Puszczalowska-Lizis E Krawczyk K Omorczyk J. Effect of longitudinal and transverse foot arch on the position of the hallux and fifth toe in preschool children in the light of regression analysis. Int J Environ Res Public Health. (2022) 19(3):1669. 10.3390/ijerph19031669 35162692 PMC8835223

[18] Bito T Tashiro Y Suzuki Y Kawagoe M Sonoda T Nakayama Y Forefoot transverse arch height asymmetry is associated with foot injuries in athletes participating in college track events. J Phys Ther Sci. (2018) 30(8):978–83. 10.1589/jpts.30.978 30154585 PMC6110222

[19] Zhao C Chen J Deng Y Huang W Ma S Su S Arch volume: a new method for medial longitudinal arch measurement. Foot Ankle Surg. (2022) 28(7):962–7. 10.1016/j.fas.2022.01.007 35105517

[20] Yamashita T Yamashita K Sato M Kawasumi M Ata S. Analysis of skeletal characteristics of flat feet using three-dimensional foot scanner and digital footprint. Biomed Eng Online. (2022) 21(1):56. 10.1186/s12938-022-01021-7 35945533 PMC9361570

[21] Wang J Tang L Tang J Chen J Gong X Qin L The typically developing pediatric foot—the data of the 1744 children in China. Foot Ankle Surg. (2022) 28(3):347–53. 10.1016/j.fas.2021.04.005 33903004

[22] Jastifer JR Toledo-Pereyra LH. Leonardo da Vinci's Foot: historical evidence of concept. J Invest Surg. (2012) 25(5):281–5. 10.3109/08941939.2012.725011 23020268

[23] Mahieu C Salvia P Beyer B Rooze M Feipel V Van Sint Jan S. Metatarsal arch deformation and forefoot kinematics during gait in asymptomatic subjects. Int Biomech. (2019) 6(1):75–84. 10.1080/23335432.2019.1642142 34042007 PMC7857307

[24] Kudo S Hatanaka Y Naka K Ito K. Flexibility of the transverse arch of the forefoot. J Orthop Surg (Hong Kong). (2014) 22(1):46–51. 10.1177/230949901402200113 24781613

[25] Matsushita T Tashiro Y Suzuki Y Tasaka S Matsubara K Matsubara K Association between height of the forefoot transverse arch and kinetics or kinematics of ankle joint during gait. Clin Res Foot Ankle. (2017) 5(1):227. 10.4172/2329-910X.1000227

[26] Górna S Pazdro-Zastawny K Basiak-Rasała A Kolator M Krajewska J Zatoński T. Characteristics of paediatric foot arches according to body mass among primary school students in wrocław, Poland. BMC Pediatr. (2022) 22(1):656. 10.1186/s12887-022-03699-z 36357927 PMC9648018

[27] Bardyugov PS Parshikov MV. Conservative correction of the transverse arch of the foot in patients with flat foot. N N Priorov J Traumatol and Orthoped. (2020) 27(2):50–9. 10.17816/vto202027250-59

